# Translationally Controlled Tumor-Associated Protein

**DOI:** 10.1155/2012/432590

**Published:** 2012-06-18

**Authors:** Malgorzata Kloc, Jacek Z. Kubiak, Rafik Mark Ghobrial

**Affiliations:** ^1^The Methodist Hospital and The Methodist Hospital Research Institute, 6670 Bertner Avenue, Houston, TX 77030, USA; ^2^CNRS UMR 6290, Institut Génétique et Développement de Rennes (IGDR) and Cell Cycle Group, Faculty of Medicine, Université de Rennes 1, 35043 Rennes, France; ^3^Department of Surgery, The Methodist Hospital, 6550 Fannin Street, Houston, TX 77030, USA

Translationally-controlled tumor-associated protein (TCTP) has been discovered in 1983 in mouse erythroleukemia cells. Over the years, it became evident that TCTP is an important player in a number of basic cell physiology events in cancer, embryo development, cell cycle, apoptosis, proliferation, growth, stress response, allergy, gene regulation, and heat-shock response. However, despite the nearly three decades of research, we only start to understand the role of TCTP in physiology of animal and plant embryo development as well as in numerous pathologies through its participation in cell cycle, proliferation, and growth regulation. The exact roles of TCTP in many complex cellular processes still remain a mystery. One of the key questions in cancer research is the role of TCTP in tumor reversion, the rare event leading to tumor regression and a “miraculous” cure: is TCTP involved in gene regulation or rather modification of the cytoskeleton of cancer cells during this process? It seems plausible that a novel type of posttranslational modification of TCTP, such as SUMOylation, by regulating its nuclear localization and/or its association with the centrosomes (both subjects featured in this issue) is responsible for some of the TCTP functions in normal and cancer cells. From presented in this issue very comprehensive and up-to-date reviews on TCTP functions, it clearly transpires that TCTP has a potential to be a crucial target for anticancer therapies. However, more research on the regulation of TCTP and its involvement in various molecular and cellular pathways and its association with subcellular structures is needed for the improvement of our understanding of this oncogene and the development of novel TCTP-targeted cancer therapies. We hope that our special TCTP issue will help in stimulation of scientific research in this field.

*Malgorzata Kloc* *Jacek Z. Kubiak* *Rafik Mark Ghobrial*

